# Genome-Wide Identification and Expression Analysis of the Cation Diffusion Facilitator Gene Family in Turnip Under Diverse Metal Ion Stresses

**DOI:** 10.3389/fgene.2018.00103

**Published:** 2018-04-04

**Authors:** Xiong Li, Yuansheng Wu, Boqun Li, Wenqi He, Yonghong Yang, Yongping Yang

**Affiliations:** ^1^Key Laboratory for Plant Diversity and Biogeography of East Asia, Kunming Institute of Botany, Chinese Academy of Sciences, Kunming, China; ^2^China Germplasm Bank of Wild Species, Kunming Institute of Botany, Chinese Academy of Sciences, Kunming, China; ^3^Key Laboratory of Agro-Biodiversity and Pest Management of Education Ministry of China, Yunnan Agricultural University, Kunming, China; ^4^College of Plant Protection, Yunnan Agricultural University, Kunming, China; ^5^College of Biology and Environmental Sciences, Jishou University, Jishou, China

**Keywords:** phylogenetic analysis, protein domain, metallic element, ion transport, gene expression

## Abstract

The cation diffusion facilitator (CDF) family is one of the gene families involved in metal ion uptake and transport in plants, but the understanding of the definite roles and mechanisms of most *CDF* genes remain limited. In the present study, we identified 18 candidate *CDF* genes from the turnip genome and named them *BrrMTP1.1*–*BrrMTP12*. Then, we performed a comparative genomic analysis on the phylogenetic relationships, gene structures and chromosome distributions, conserved domains, and motifs of turnip CDFs. The constructed phylogenetic tree indicated that the BrrMTPs were divided into seven groups (groups 1, 5, 6, 7, 8, 9, and 12) and formed three major clusters (Zn-CDFs, Fe/Zn-CDFs, and Mn-CDFs). Moreover, the structural characteristics of the BrrMTP members in the same group were similar but varied among groups. To investigate the potential roles of BrrMTPs in turnip, we conducted an expression analysis on all *BrrMTP* genes under Mg, Zn, Cu, Mn, Fe, Co, Na, and Cd stresses. Results showed that the expression levels of all *BrrMTP* members were induced by at least one metal ion, indicating that these genes may be related to the tolerance or transport of those metal ions. Based on the roles of different metal ions for plants, we hypothesized that *BrrMTP* genes are possibly involved in heavy metal accumulation and tolerance to salt stress apart from their roles in the maintenance of mineral nutrient homeostasis in turnip. These findings are helpful to understand the roles of MTPs in plants and provide preliminary information for the study of the functions of *BrrMTP* genes.

## Introduction

Normal plant growth requires major and trace elements containing metallic elements, such as K, Ca, Mg, Fe, Na, Cu, Zn, Mn, Ni, and Co, which play various roles in plants. However, these trace elements are highly toxic at excessive amounts. Meanwhile, several non-essential and toxic metal ions, including Cd, Pb, and Hg, have the same chemical structures as some of these trace elements and are thus absorbed by plants (Kim et al., 2004). Salt stress and heavy metal toxicity commonly occur because of the excessive accumulation of various metal ions. Conversely, trace metal element deficiency in poor soils decreases agricultural yield and intake of trace elements in the human body. Therefore, precise metallic element homeostasis is essential in plants (Kim et al., 2004). Several gene families participate in the uptake and transport of metal ions by plants (Lang et al., 2011; Zhang et al., 2013). These metal ion transporters include the cation diffusion facilitator (CDF) family, Zrt/Irt-like protein, P-type ATPase, and ATP-binding cassette transporter, etc., (Zhang et al., 2013).

The CDF family, also called the cation efflux family (Lang et al., 2011), was first described in 1995 (Nies and Silver, 1995). The protein members of the CDF family have been identified in all six kingdoms of living things (Migocka et al., 2015a) and are involved in transport and tolerance to trace elements (Kim et al., 2004). CDF family members have three distinct features, namely, an N-terminal signature sequence, cation efflux domain, and approximately six predicted transmembrane regions (Paulsen and Saier, 1997). Eukaryotic CDFs usually contain a histidine (His)-rich cytoplasmic loop between transmembrane domains 4 and 5 (Paulsen and Saier, 1997). CDF members are classified into three major clusters, namely, Zn-CDFs, Fe/Zn-CDFs, and Mn-CDFs, which differ with respect to selectivity toward the main transported metal ions (Montanini et al., 2007). In plants, CDF members are designated as metal tolerance proteins (MTPs; Migocka et al., 2015b). The MTP family has 12 and 10 members in model plants *Arabidopsis* and rice, respectively. Unfortunately, their functions remain finitely understood (Migocka et al., 2015b). Recently, MTP proteins from different plant species have been divided into seven groups according to the results of the phylogenetic analysis and annotation performed on *Arabidopsis* MTPs (Gustin et al., 2011). Among them, groups 1, 5, and 12 belong to the Zn-CDFs, groups 6 and 7 form the Fe/Zn-CDFs, and the Mn-CDF cluster comprises groups 8 and 9 (Gustin et al., 2011). To date, the most studied MTP proteins in plants include the MTP1–MTP4 members of group 1, MTP8 proteins of group 8, and MTP9–MTP11 of group 9 (Migocka et al., 2015b). Most of these MTPs were functionally characterized in diverse plant species. Although group 1 of plant MTPs belongs to Zn-CDFs, its members is capable of transporting different metals, including Zn, Cd, Co, Ni, or Fe, into the vacuole of plant cells (Kim et al., 2004; Xu et al., 2009; Lang et al., 2011; Menguer et al., 2013; Migocka et al., 2015a). Recently, MTP5 has been reported to form a functional complex with MTP12 to transport Zn into the Golgi apparatus in *Arabidopsis* (Fujiwara et al., 2015). Group 8, comprising only MTP8 proteins, is implicated in Mn homeostasis, which requires a proton gradient when transporting Mn^2+^ across membranes (Migocka et al., 2015b; Eroglu et al., 2017; Li Q.H. et al., 2017). They are necessary for the active sequestration of excess Mn in intracellular organelles or Mn delivery to Mn-dependent enzymes (Migocka et al., 2015b). Meanwhile, group 9 comprises MTP9–MTP11 proteins (Montanini et al., 2007; Gustin et al., 2011), and MTP 9 and MTP 10 are more closely related and thus form an internal separate clade. This relationship indicates some functional differentiation between MTP9/10 and MTP11 proteins (Gustin et al., 2011). Several MTP11 transporters from different species confer Mn^2+^ tolerance (Delhaize et al., 2007; Zhang and Liu, 2017), whereas limited evidence of the involvement of MTP9/10 proteins in Mn transport or tolerance to Mn is currently available (Migocka et al., 2015b), though they are also classified as Mn-CDFs. Current studies generally indicated that MTP proteins contribute to the detoxification of heavy metals and promote their accumulation (Kim et al., 2004; Delhaize et al., 2007; Xu et al., 2009; Lang et al., 2011; Menguer et al., 2013; Migocka et al., 2015a,b; Eroglu et al., 2017; Li Q.H. et al., 2017; Zhang and Liu, 2017). The functions of MTPs or other metal transporters in heavy metal hyperaccumultors may improve phytoremediation efficiency (Kim et al., 2004; Gustin et al., 2009). However, knowledge on the functional assignment of plant MTPs remains insufficient. For example, the functional characteristics of groups 6 and 7 have not been elucidated so far.

The *Brassicaceae* species has shown apparent advantages in the exploration of metal ion transporter functions. These species include the model plant *Arabidopsis* and many important vegetables and oilseed crops with easily available genomes (Wang et al., 2011; Liu et al., 2014; Kolaj-Robin et al., 2015; Gan et al., 2016; Yang et al., 2016), and have represented a differential range of tolerance to two important abiotic stresses, namely, salinity and heavy metals (Assunção et al., 2003; Prasad and Freitas, 2003; Inan et al., 2004; Megdiche et al., 2007). For example, over half of the identified Ni hyperaccumulators (more than 300 species) were reportedly from *Brassicaceae* (Assunção et al., 2003). Turnip (*Brassica rapa* var. *rapa*), a *Brassicaceae* biennial plant, has been widely cultivated in Europe, Asia, and America as a vegetable or fodder. In Asia, one of the cultivation centers of turnip is in the Tibetan Plateau and its surrounding countries and regions. Moreover, turnip has been cultivated at the maximum elevation of 4700 m in Nyima County, China. Thus, this species is faced with diverse soil conditions, indicating that turnips from different populations or cultivated landraces may possess various ion absorption or accumulation characteristics to adapt to the corresponding environments. Turnips from Xinjiang, China, reportedly contain abundant mineral elements (Ma et al., 2016), indicating its eminent absorption ability for metal ions. Additionally, turnip has been classified as a high-Cd accumulation plant (Arthur et al., 2000; Li et al., 2016). We found that turnip landraces from China have strong capacities for Cd accumulation, and several landraces were Cd hyperaccumulators (Li et al., 2016, Li X. et al., 2017). To explore the molecular mechanism of metal ion accumulation in turnip, we focused on several important ion transporters based on the sequencing results of turnip (Cheng et al., 2016). In the present study, we systematically analyzed the sequence and structural characteristics of putative MTPs in turnip and preliminarily investigated the potential roles of each BrrMTP member. This study is expected to improve the understanding of the functions of plant MTPs and provide a basis for the analysis of the functions and mechanisms of BrrMTP proteins.

## Materials and Methods

### Identification and Phylogenetic Analysis of the MTPs in Turnip

The gene sequences of 12 *MTP*s in *Arabidopsis* were downloaded from NCBI^1^ as queries to search against the turnip genome ^2^. The domains and functional sites in each protein were examined with InterProScan^3^ (Finn et al., 2017). All protein sequences containing any of the typical domains of MTP proteins were extracted as candidates. The candidates were then used to search against the GenBank non-redundant protein database. The ClustalW software was used for the sequence alignment between turnip and *Arabidopsis*, and phylogenetic trees were constructed using the MEGA 7.0 software. The neighbor-joining method was performed and 1000 bootstrap test replicates were used during the construction (Kumar et al., 2016).

### Gene Structure and Location on Chromosomes of Turnip *MTP* Genes

The diagram of the intron/exon structures of *BrrMTP* genes was analyzed by using the online Gene Structure Display Server^4^ (Hu et al., 2015). The chromosomal location of the *BrrMTP* genes was mapped according to the gene position information using the TBtools^5^.

### Structure Characteristics and Physicochemical Parameters of Turnip MTP Proteins

The Pfam tool^6^ and the MEME^7^ program were used to search for conserved domains and motifs in the BrrMTP protein sequences, respectively (Sonnhammer et al., 1998; Bailey et al., 2006); subsequently, the domain and motif diagrams were drawn with the TBtools software. The putative transmembrane regions in proteins were predicted by using the TMHMM Server V. 2.0^8^. The physicochemical parameters of the proteins, including molecular weight (MW), theoretical isoelectric point (pI), and grand average of hydropathicity (GRAVY), were calculated with the ProtParam tool of ExPaSy^9^ (Artimo et al., 2012).

### Plant Growth and Stress Treatments

Turnip seeds were sown in soil pots under the natural condition. After 2 weeks of growth, the turnip seedlings were transplanted into uniform pots (*d* = 9.4 cm, *h* = 8 cm) with uniform soil (one seedling in a pot) under greenhouse condition (22–25°C, 12-h light/12-h darkness, 50–60% relative humidity). A total of 3 weeks later, the plants were irrigated with eight metal ion solutions. The plant irrigated with water only was used as the control. Each pot was irrigated with a 100 mL solution, and the pots were placed in culture dishes. The ion sources and concentrations are shown in **Table 1**. The roots and leaves of each treatment were harvested separately, ground to powder and then stored at –80°C for RNA isolation. Three biological replicates were made for each treatment.

**Table 1:** Concentrations and sources of different metal ions used to treat turnip.

Metal ion	Concentration (mg L^-1^)	Source
Mg	10.0	MgCl_2_⋅6H_2_O
Zn	2.0	ZnSO_4_⋅7H_2_O
Cu	5.0	CuSO_4_⋅5H_2_O
Mn	5.0	MnSO_4_
Fe	2.5	FeCl_2_⋅4H_2_O
Co	2.0	CoCl_2_⋅6H_2_O
Na	2.5	NaCl
Cd	2.0	CdCl_2_⋅2.5H_2_O

### RNA Extraction and cDNA Synthesis

The total RNA samples were isolated using the Eastep^®^ Super Total RNA Extraction Kit (Promega, Madison, WI, United States). The RNA concentration was determined by NanoDrop1000 (NanoDrop Technologies, Inc.), with the integrity checked on 0.8% agarose gel. A total of 3 μg of RNA was reverse-transcribed using the GoScript Reverse Transcription System (Promega, Madison, WI, United States) to generate the cDNA.

### Quantitative Real-Time PCR (qRT-PCR) Analysis

Optimal forward and reverse primers were designed (Supplementary Table S1) through the online tool Primer-BLAST^10^ for qRT-PCR analysis. qRT-PCR was conducted in triplicate with different cDNAs from different tissues and treatments. FastStart Universal SYBR Green Master (Rox, Roche, Indianapolis, IN, United States) and a 7500 Sequence Detection System (Applied Biosystems, United States) were used. The reaction parameters for thermal cycling were as follows: 95°C for 10 min, followed by 40 cycles of 94°C for 5 s, 60°C for 15 s and 72°C for 34 s. The turnip beta-tubulin gene was amplified as an internal control. The relative gene expression levels were obtained by dividing the extrapolated transcript levels of the target genes by the levels of the internal control from the same sample.

### Statistical Analysis

Statistical analyses were performed using SPSS version 18.0. One-way ANOVA or independent-samples *t*-test was conducted to analyze significant differences among multiple samples or between each pair of samples at a 0.05 level, respectively.

## Results

### Identification and Phylogeny of the MTPs in Turnip

**FIGURE 1:**
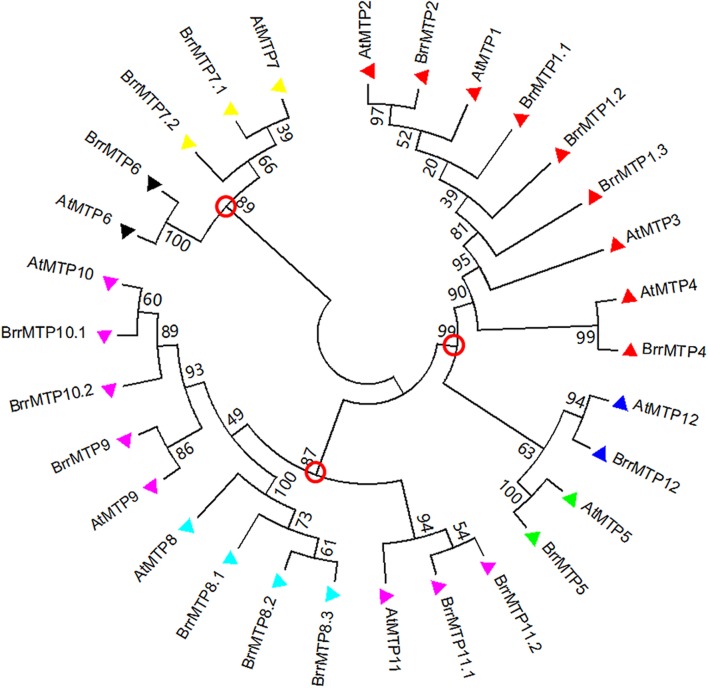
Phylogenetic relationship of MTP proteins in turnip and *Arabidopsis*. The protein sequences were aligned by ClustalW, and phylogenetic trees were constructed using the MEGA 7.0 software with the neighbor-joining method and 1000 bootstrap test. The red circles labeled on the branch roots indicate the divided three clusters of MTP proteins. Triangles with different colors represent the seven groups of MTP proteins (red: group 1; green: group 5; black: group 6; yellow: group 7; light blue: group 8; pink: group 9; blue: group 12).

We identified 18 MTPs in turnip on the basis of the published genome result (**Table 2**). The MTP family in turnip was more expanded than that in *Arabidopsis*. The complete coding region, CDS, and protein sequences of all turnip MTPs were identified for subsequent information analysis. To gain insights into the phylogenetic relationship of the MTPs between turnip and *Arabidopsis*, we used the MTP family protein sequences to build a phylogenetic tree. According to the orthologous relationships, the turnip MTPs were designated as BrrMTP1.1 to BrrMTP12 (**Figure 1**). Interestingly, we found multiple homologous *AtMTP1*, *AtMTP7*, *AtMTP8*, *AtMTP10*, and *AtMTP11* genes in the turnips, whereas no *AtMTP3* orthologous gene was found (**Figure 1**). Accordingly, the 18 BrrMTPs were divided into seven groups (i.e., groups 1, 5, 6, 7, 8, 9, and 12), which formed the three clusters of the phylogenetic tree, namely, Zn-CDFs, Fe/Zn-CDFs, and Mn-CDFs.

**Table 2:** Turnip *MTP* genes identified and their characteristics.

Gene	Gene locus	Coding region length (bp)	CDS length (bp)	Protein size (aa)	MW (kD)	pI	GRAVY
*BrrMTP1.1*	A03:11044471..11045628	1158	1158	385	42.44	5.91	0.190
*BrrMTP1.2*	A04:18865311..18866459	1149	1149	382	42.03	5.81	0.251
*BrrMTP1.3*	A05:480592..481722	1131	1131	376	41.50	5.69	0.242
*BrrMTP2*	A09:30174575..30175582	1008	1008	335	37.51	6.15	0.331
*BrrMTP4*	A04: 13183256..13185186	1931	1131	376	42.52	5.81	0.086
*BrrMTP5*	A05:21290583..21293550	2968	1173	390	43.50	6.87	0.193
*BrrMTP6*	A03:11190449..11192972	2524	1395	464	50.52	7.44	0.004
*BrrMTP7.1*	A05:11112916..11115625	2710	1362	453	49.94	7.76	-0.031
*BrrMTP7.2*	A06:1264317..1267227	2911	1374	457	49.95	8.22	-0.039
*BrrMTP8.1*	A09:28826687..28828553	1867	1233	410	46.39	5.25	0.024
*BrrMTP8.2*	A04:1622033..1623914	1882	1218	405	45.58	5.01	0.097
*BrrMTP8.3*	A07:12509876..12511731	1856	1233	410	46.21	5.46	0.078
*BrrMTP9*	A07:22190959..22193339	2381	1293	430	48.87	7.05	-0.119
*BrrMTP10.1*	A06:6239593..6241846	2254	1362	453	52.25	6.86	-0.125
*BrrMTP10.2*	A09:33327854..33330419	2566	1206	401	45.62	6.14	-0.081
*BrrMTP11.1*	A05:3062557..3064672	2116	1176	391	44.31	5.18	0.045
*BrrMTP11.2*	A04:16758980..16760595	1616	1185	394	44.59	5.07	0.005
*BrrMTP12*	A04:7455917..7458214	2298	2298	765	86.24	6.74	0.022

### Gene Structure and Location of Turnip *MTP* Genes

**FIGURE 2:**
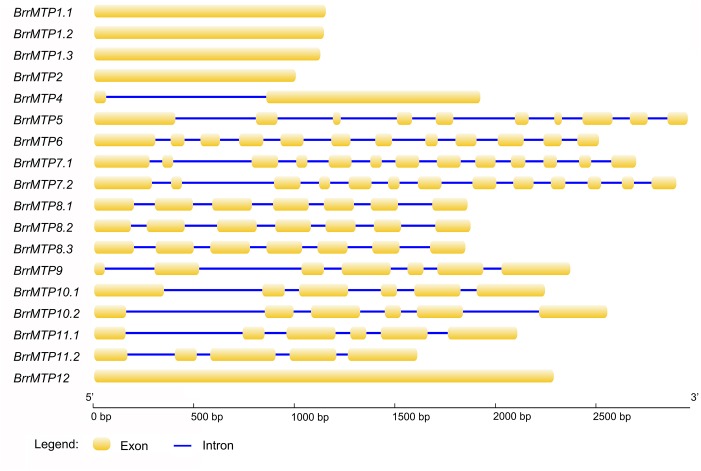
Gene structures of the coding regions of the turnip *MTP* genes.

The lengths of the coding region sequences (including exons and introns) of the turnip *MTP* genes ranged from 1008 bps (*BrrMTP2*) to 2968 bps (*BrrMTP5*) (**Table 2**), and their CDS sequences included 1008–2298 bps (**Table 2**), which encoded 335–765 amino acids (**Table 2**). The comparative analysis between the CDS and the gene sequences indicated that the *BrrMTPs* of the three clusters contained differential introns (**Figure 2**). The *Zn-CDFs* contained 0 or 1 intron, except for *BrrMTP5* (11), and the *Fe/Zn-CDFs* included 11 or 12 introns, whereas the members of *Mn-CDFs* had 4–6 introns (**Figure 2**).

**FIGURE 3:**
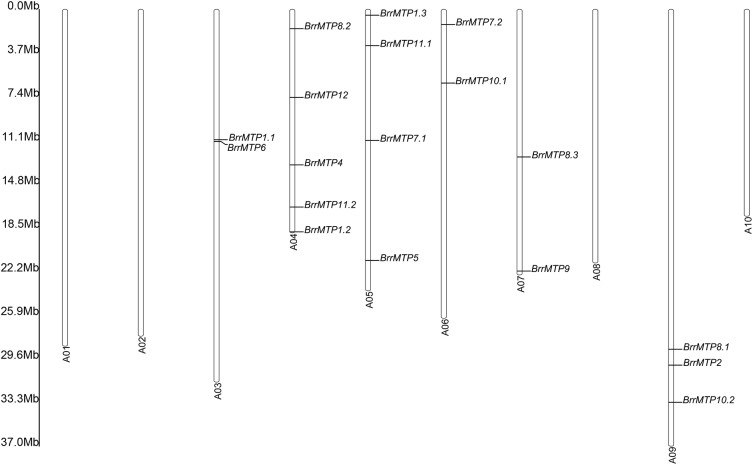
Distribution of the *MTP* genes on turnip chromosomes. A01∼A10 represent the chromosome No, and the rule on the left indicates the physical map distance among genes (Mb).

The chromosomal location showed that the 18 *BrrMTP* genes were located in 6 out of the 10 turnip chromosomes (**Figure 3**). Among them, chromosome A04 contained the maximum number of 5 *BrrMTP* genes (**Figure 3**). Chromosomes A03, A05, A06, A07, and A09 contained 2–4 *BrrMTPs*, whereas chromosomes A01, A02, A08, and A10 carried no *BrrMTP* genes (**Figure 3**).

### Structure Characteristics and Physicochemical Parameters of Turnip MTP Proteins

**FIGURE 4:**
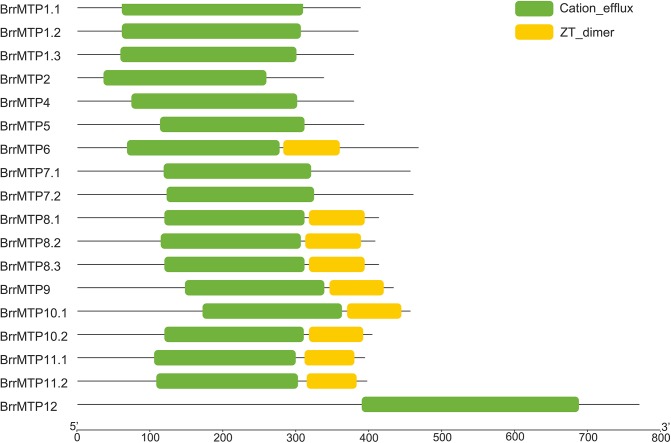
Distribution of the conserved structural domains in turnip MTP proteins.

Protein structure analysis showed that all the BrrMTPs contained the cation efflux domain, while the members of groups 6, 8, and 9 had a common ZT dimer structure (**Figure 4**). Other features of MTP proteins were also observed in different members of *BrrMTPs*. The CDF signature sequence was observed in the N-terminals of the group 1 BrrMTPs (**Table 3** and Supplementary Figure S1). Most BrrMTP proteins contained 4–6 typical transmembrane regions, except for BrrMTP12, which contained 14, and BrrMTP6, which possibly contained 4 (**Table 3**). Additionally, the BrrMTP members of groups 1 and 12 contained a His-rich region (**Table 3** and Supplementary Figure S1). We further analyzed the diversity of the conserved motif compositions in BrrMTPs using the MEME program. A total of 20 conserved motifs, designated as motifs 1–20, were identified within the proteins (Supplementary Figure S2). The members of the same cluster (or the same group) generally contained similar motifs (Supplementary Figure S2). Each cluster included several relatively specific motifs, while motif 4 was shared by all BrrMTPs, except for BrrMTP 5, which only contained two instances of motif 8 (Supplementary Figure S2). Most BrrMTPs contained one or two duplicates of the same motif, whereas BrrMTP12 had five duplicates of motif 15 (Supplementary Figure S2).

**Table 3:** Number of conserved features in turnip MTP proteins.

Cluster	Group	Protein	CDF signature	Transmembrane region	Histidine rich region
Zn-CDFs	Group 1	BrrMTP1.1	1	6	1
		BrrMTP1.2	1	6	1
		BrrMTP1.3	1	6	1
		BrrMTP2	1	6	1
		BrrMTP4	1	5	1
	Group 5	BrrMTP5	0	6	0
	Group 12	BrrMTP12	0	14	1
Fe/Zn-CDFs	Group 6	BrrMTP6	0	(4)	0
	Group 7	BrrMTP7.1	0	4	0
		BrrMTP7.2	0	4	0
Mn-CDFs	Group 8	BrrMTP8.1	0	5	0
		BrrMTP8.2	0	4	0
		BrrMTP8.3	0	5	0
	Group 9	BrrMTP9	0	4	0
		BrrMTP10.1	0	4	0
		BrrMTP10.2	0	5	0
		BrrMTP11.1	0	5	0
		BrrMTP11.2	0	5	0

*The value inside the brackets indicates a possible result*.

The physicochemical parameters, including the MW, pI, and GRAVY values, of the BrrMTP proteins were predicted as shown in **Table 2**. The MW values ranged from 37.51 kD (BrrMTP2) to 86.24 kD (BrrMTP12), most of which were within 40–50 kD (**Table 2**). Nearly all the pI values of all BrrMTPs of Zn-CDFs and Mn-CDFs were lower than 7.0, whereas those of the members of Fe/Zn-CDFs were higher than 7.0 (**Table 2**). The GRAVY results, ranging from -0.119 (BrrMTP9) to 0.331 (BrrMTP2), indicated that the BrrMTPs generally had weak hydrophilicity (**Table 2**).

### Expression Profiles of Turnip *MTP* Genes in Different Tissues

**FIGURE 5:**
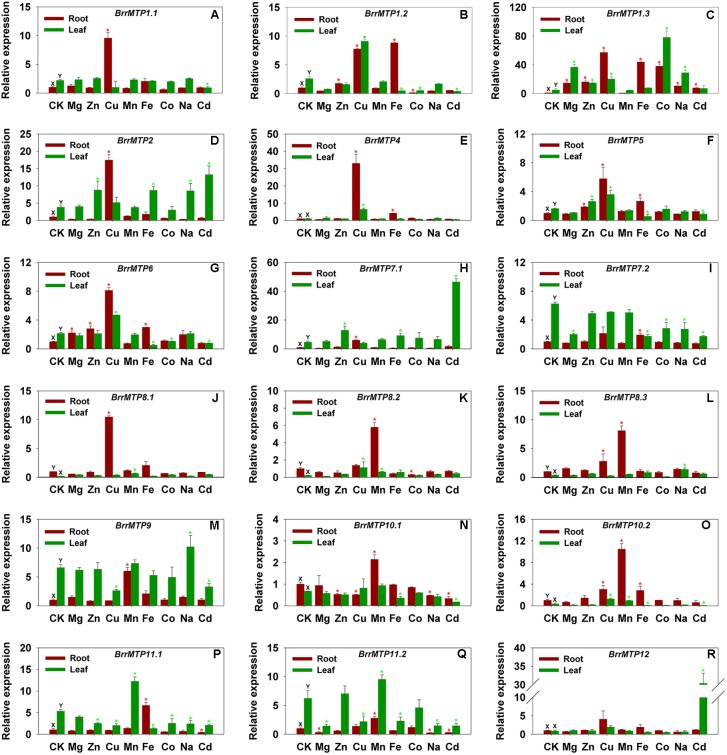
Relative expression levels of turnip *MTP* genes under various metal ion stresses in root or leaf. Data represent means ± SD **(A–R)**. CK represents control samples **(A–R)**. Different letters (X and Y) indicate significant differences between root and leaf under normal condition (*n* = 3, *P* < 0.05) **(A–R)**. Red and green asterisks indicate significant differences between the treatment samples and the control samples in root and leaf, respectively (*n* = 3, *P* < 0.05) **(A–R)**.

The qRT-PCR results indicated that the *BrrMTP* genes had differential tissue expression patterns at the seedling stage in soil. We found that the expressions of *BrrMTP1.1*, *BrrMTP1.2*, *BrrMTP1.3*, *BrrMTP2*, *BrrMTP5*, *BrrMTP6*, *BrrMTP7.1*, *BrrMTP9*, *BrrMTP11.1*, and *BrrMTP11.2* in the turnip leaves were much higher than those in the roots (*P* < 0.05), whereas the expressions of *BrrMTP8.1*, *BrrMTP8.2*, *BrrMTP8.3*, and *BrrMTP10.2* showed the opposite results (**Figure 5**). *BrrMTP4*, *BrrMTP10.1*, and *BrrMTP12* showed similar expression levels between roots and leaves (**Figure 5**). Interestingly, the homologous genes broadly showed similar tissue expression characteristics (**Figure 5**).

### Expression Differences of Turnip *MTP* Genes Under Diverse Metal Ion Stresses

To explore the potential roles of the *BrrMTP* genes, we analyzed expression changes in the entire *MTP* family of turnips in the presence of different metal ions, including macroelements (e.g., Mg), microelements (e.g., Zn, Cu, Mn, Fe, Co, and Na) and non-essential elements (e.g., Cd). The *BrrMTP* genes showed various responses to the same metal ion either in the root or leaf (**Figure 5**), and the expression of a certain gene differentially changed under different metal treatments (**Figure 5**). We summarized the metal ions that significantly induce or inhibit the expression of each *BrrMTP* gene in either root or leaf (*P* < 0.05). Each metal ion used in the experiment induced the expression levels of one or several *BrrMTP* genes in the root or leaf (**Figure 5** and **Table 4**). In detail, Mg induced the expression levels of *BrrMTP1.3* and *BrrMTP6* (**Figure 5** and **Table 4**); Zn induced the expression levels of *BrrMTP1.2*, *BrrMTP1.3*, *BrrMTP2*, *BrrMTP5*, *BrrMTP6*, and *BrrMTP7.1* (**Figure 5** and **Table 4**); Cu induced the expression levels of *BrrMTP1.1*, *BrrMTP1.2*, *BrrMTP1.3*, *BrrMTP2*, *BrrMTP4*, *BrrMTP5*, *BrrMTP6*, *BrrMTP7.1*, *BrrMTP8.1*, *BrrMTP8.3*, and *BrrMTP10.2* (**Figure 5** and **Table 4**); Mn induced the expression levels of all Mn-CDFs (*BrrMTP8.1*–*BrrMTP11.2*; **Figure 5** and **Table 4**); Fe induced the expression levels of *BrrMTP1.2*, *BrrMTP1.3*, *BrrMTP2*, *BrrMTP4*, *BrrMTP5*, *BrrMTP6*, *BrrMTP7.1*, *BrrMTP7.2*, *BrrMTP10.2*, and *BrrMTP11.1* (**Figure 5** and **Table 4**); Co only induced the expression of *BrrMTP1.3* (**Figure 5** and **Table 4**); Na induced the expression levels of *BrrMTP1.3*, *BrrMTP2*, *BrrMTP8.3*, and *BrrMTP9* (**Figure 5** and **Table 4**); and Cd induced the expression levels *BrrMTP1.3*, *BrrMTP2*, *BrrMTP7.1*, and *BrrMTP12* (**Figure 5** and **Table 4**). Meanwhile, the *BrrMTP* genes showed upregulated expressions under at least one metal ion treatment (**Figure 5** and **Table 4**). In particular, the expression of BrrMTP 1.3 was upregulated under seven metal ion treatments (**Figure 5** and **Table 4**). On the contrary, the expression levels of *BrrMTP1.1*, *BrrMTP1.2*, *BrrMTP5*, *BrrMTP6*, *BrrMTP7.2*, *BrrMTP8.2*, *BrrMTP9*, *BrrMTP10.1*, *BrrMTP10.2*, *BrrMTP11.1*, and *BrrMTP11.2* were downregulated under a single or several metal ion treatments in the roots or leaves and were especially inhibited by Cd (**Figure 5** and **Table 4**). Generally, although the genes of the same group, even the homologous genes, did not show obviously similar expression changes to the same metal ion, the same cluster B*rrMTP* genes showed some common characteristics (**Figure 5** and **Table 4**). For example, the members of *Zn-CDFs* showed a common positive response to several metals, including Zn, Cu, Fe, and Cd, whereas the Mn-CDF genes were mainly induced by the Mn ion but widely inhibited by other metal ions (**Figure 5** and **Table 4**).

**Table 4:** Summary of the metal ions that either induce or inhibit the expressions of the turnip *MTP* genes.

Cluster	Group	Gene	Metal ions that induce gene expression		Metal ions that inhibit gene expression	
			In root	In leaf	In root	In leaf
Zn-CDFs	Group 1	*BrrMTP1.1*	Cu	–	–	Cd
		*BrrMTP1.2*	Zn, Cu, Fe	Cu	Co	Fe, Co, Cd
		*BrrMTP1.3*	Mg, Zn, Cu, Fe, Co, Na, Cd	Mg, Zn, Cu, Co, Na	–	–
		*BrrMTP2*	Cu	Zn, Fe, Na, Cd	–	–
		*BrrMTP4*	Cu, Fe	Cu	–	–
	Group 5	*BrrMTP5*	Zn, Cu, Fe	Zn, Cu	–	Fe, Cd
	Group 12	*BrrMTP12*	–	Cd	–	–
Fe/Zn-CDFs	Group 6	*BrrMTP6*	Mg, Zn, Cu, Fe	Cu	–	Fe, Co, Cd
	Group 7	*BrrMTP7.1*	Cu	Zn, Fe, Cd	–	–
		*BrrMTP7.2*	Fe	–	–	Mg, Fe, Co, Na, Cd
Mn-CDFs	Group 8	*BrrMTP8.1*	Cu	Mn	–	–
		*BrrMTP8.2*	Mn	Cu, Mn	Co	–
		*BrrMTP8.3*	Cu, Mn	Na	–	–
	Group 9	*BrrMTP9*	Mn	Na	–	Cu, Cd
		*BrrMTP10.1*	Mn	–	Zn, Cu, Na, Cd	Fe, Cd
		*BrrMTP10.2*	Cu, Mn, Fe	Cu, Mn	–	Fe, Cd
		*BrrMTP11.1*	Fe	Mn	Cd	Zn, Cu, Fe, Co, Na, Cd
		*BrrMTP11.2*	Mn	Mn	Na, Cd	Mg, Cu, Fe, Na, Cd

## Discussion

Although increasing functional studies on plant *MTP* members have been reported in diverse species, such as *Arabidopsis thaliana* (Desbrosses-Fonrouge et al., 2005; Delhaize et al., 2007; Eroglu et al., 2016, 2017), *Oryza sativa* (Menguer et al., 2013; Zhang and Liu, 2017), *Brassica juncea* (Xu et al., 2009; Lang et al., 2011), *Cucumis sativus* (Migocka et al., 2015a,b), *Thlaspi goesingense* (Kim et al., 2004), and *Camellia sinensis* (Li Q.H. et al., 2017), the understanding of the roles of the *MTP* family is still significantly limited. On the basis of the increasing genome information, analyzing gene families via comparative genomics is an efficient method for modern functional genomics research (Xu et al., 2016). However, unlike many other gene families, including some transcription factors and kinases (Iftikhar et al., 2017; Ma et al., 2017; Xi et al., 2017), studies on the characteristics and functional analysis of the plant *MTP* gene family are just reported in few species recently (Fu et al., 2017; Vatansever et al., 2017). As *MTP* genes have been demonstrated to participate in tolerating and transporting various heavy metals, including plant trace elements and non-essential elements (Kim et al., 2004; Delhaize et al., 2007; Xu et al., 2009; Lang et al., 2011), they might thus play significant roles in plant mineral nutrition maintenance and resistance to stresses caused by metals. Turnip has shown relatively high capacities toward absorbing trace elements and heavy metal Cd (Li et al., 2016; Ma et al., 2016). Thus, exploring the functional characteristics of the metal ion transporters in turnip is of great interest. In the present study, 18 MTP family members in turnip were successfully identified by bioinformatics analysis. These 18 *BrrMTPs* were located at different positions on 6 chromosomes and were unevenly distributed within the genome. Compared with *Arabidopsis*, gene family expansion occurred in the *BrrMTP* gene family. This might be because the polyploidization events occurred in the evolutionary history of *Brassica rapa* (Yin et al., 2017). These events were followed by chromosomal reduction and rearrangement and numerous gene losses (Yin et al., 2017). In the present results, *MTP3* was not observed in turnip; this was likely because of gene loss. However, the incomplete genomes factor was not excluded. The increase in the number of *MTP* genes during plant evolution was likely related to the functional evolution of metal tolerance and accumulation.

The phylogenetic relationship indicated that BrrMTPs could be divided into three clusters and seven groups as in *Arabidopsis* (Montanini et al., 2007; Kolaj-Robin et al., 2015). Clusters Zn-CD*F*, Fe/Zn-CDF, and Mn-CDF contained 7, 3, and 8 members, respectively. This classification was supported by the subsequent analysis of gene structure and protein features. Although the coding regions of most *BrrMTPs* (except *BrrMTP12*) encoded polypeptides of 335–464 amino acids in length, the intron numbers of *BrrMTPs* had significant differences among different groups, ranging from 0 to 12. Variations in intron lengths also existed in the *BrrMTPs*. However, their functions and evolutionary process require further verification. Like the protein size, the MW, pI, and GRAVY of most BrrMTPs were relatively conserved or similar but closer within groups. Particularly, BrrMTP12 possessed significantly larger protein size (765 amino acids) and MW (86.24 kD) than the other BrrMTP members. This result was consistent with the difference between AtMTP12 and other AtMTPs, indicating the distinctive function and evolutionary process of MTP12. Undoubtedly, all the BrrMTPs contained the conserved cation efflux domain in their sequences. Moreover, a zinc transporter dimerization (ZT dimer) domain was detected in the members of groups 6, 8, and 9, but whether this domain is correlated with the functions of these BrrMTP is unknown. The CDF signature sequence, transmembrane region and His-rich loop are three significant structural features of MTP proteins (Montanini et al., 2007; Kolaj-Robin et al., 2015), which are closely related to their functional characteristics. However, these features showed obvious differences among different groups (or clusters). The complete CDF signature sequences were only observed in the BrrMTPs of group 1. The His-rich loop in the MTP protein sequences has been considered responsible for metal selectivity (Kang and Carey, 1999; Podar et al., 2012; Kolaj-Robin et al., 2015). In the present study, groups 1 and 12 from the Zn-CDF cluster possessed individual His-rich sequences. However, BrrMTP1.1–BrrMTP4 included 17–40 amino acid residues in length, whereas the sequence length of BrrMTP12 was 115 amino acid residues. The results might indicate the differential abilities of different BrrMTPs to transport metal ions. Unlike the above two features, most BrrMTPs contained 4–6 conserved transmembrane regions, except for BrrMTP12, which possessed 14 ones, similar to 15 ones in AtMTP12 (Zhang et al., 2013). Generally, these results were consistent with the structure characteristics of AtMTPs. In *Arabidopsis*, AtMTP5–AtMTP12 proteins only contained one of the features of the MTP family, such as the transmembrane domains, whereas AtMTP1–AtMTP4 included all the MTP features (Paulsen and Saier, 1997; Zhang et al., 2013). However, three different clusters were divided by the researchers, which included AtMTP6–AtMTP11 (cluster I), AtMTP5 and AtMTP12 (cluster II), and AtMTP1–AtMTP4 (cluster III) (Paulsen and Saier, 1997; Zhang et al., 2013). The results of motif compositions also showed the structural similarity within the BrrMTP groups and the particularity among different groups, indicating the possible functional diversity of the entire family of BrrMTPs.

To date, members of plant MTPs have been reported to be involved in tolerating and transporting different metals, including Zn, Cd, Co, Ni, Fe, and Mn (Kim et al., 2004; Xu et al., 2009; Lang et al., 2011; Menguer et al., 2013; Migocka et al., 2015a,b; Eroglu et al., 2017; Li Q.H. et al., 2017; Zhang and Liu, 2017). Thus, these transporters have shown potential applications in the phytoremediation of heavy metal-polluted soils, especially the members from heavy metal hyperaccumulators (Kim et al., 2004; Gustin et al., 2009). Eroglu et al. (2016) recently has reported that MTP8 in *Arabidopsis* determines tolerance to iron deficiency-induced chlorosis; this study provides a new insight into roles of MTP proteins in plants. However, whether the MTP family contributes to the plant response toward salt stress is unclear. Moreover, whether the different members have function division or cooperation when transporting various metal ions remains undefined. To expand the information for these issues, we predicted the potential substrate metal ions of each member of the BrrMTPs by performing gene-induced expression. We primarily examined the spatial expression patterns of the *BrrMTP* genes under normal nutrient conditions. The results showed that all members of groups 1, 5, 6, and 7 and several members of group 9 (*BrrMTP9*, *BrrMTP11.1* and *BrrMTP11.2*) were mainly expressed in the leaves, whereas most of the others were expressed in the roots, indicating that different *BrrMTP* members play specific roles or run different mechanisms in turnips. The results are partly consistent with the reports regarding their individual homologous genes (Lang et al., 2011; Chen et al., 2013; Vatansever et al., 2017). This might be related to the special nutrient transport during growth and development of turnips, which form fleshy roots. To investigate the potential roles of *BrrMTP* members, we detected the expression dynamics of the *BrrMTP* genes under eight metal ion treatments. These metal ions include a macroelement (Mg), microelement (Zn, Cu, Mn, Fe, Co, or Na) or non-essential element (Cd) for plant growth and also represent heavy metals (e.g., Zn, Cu, Mn, and Cd) or salt ions (e.g., Mg and Na). The results showed that each metal ion induced the expression levels of at least one *BrrMT*P gene in the roots and leaves, indicating that the *BrrMTPs* may be involved in the tolerance or transport of corresponding macroelements and microelements. In that case, the *BrrMTPs* are of significance for turnip plants to maintain mineral element balance under normal condition or to improve tolerance to heavy metals and salts when faced with stresses. Cd is a non-essential element to plants, and plants do not possess specific mechanisms for the uptake of Cd^2+^, but Cd can be absorbed and transported through some of the carriers used for the uptake of essential metals for plant development (Li L.Z. et al., 2017). We found that *BrrMTP1.3*, *BrrMTP2*, *BrrMTP7.1*, and *BrrMTP12* were increasingly expressed when treated by Cd, indicating that these genes may be related to the high-Cd accumulation characteristics of turnip (Li et al., 2016). Interestingly, our results are consistent with those of previous studies wherein the *MTPs* of group 1 were reported to transport multiple metal ions, whereas the members of group 8 or 9 showed a relative specificity to Mn (Kim et al., 2004). However, our results possibly indicated that the *MTP* genes of turnip have extended functions during the evolution or domestication process. For instance, we found that *BrrMTP1.*3, an expansion member of group 1 in turnip, showed a positive response (upregulated expression) to seven metal ions in contrast to *Arabidopsis*. We also found that several metal ions induced the expression levels of groups 6 and 7 in the roots and leaves. Given that the functions of these *MTP* genes are rarely reported, this study provided a basis for their functional investigation. Additionally, although Cu has not been identified as substrates for MTP transporters even in model plants, we found that Cu induced the expression levels of most *BrrMTP* genes. The results are supported by a recent study in sweet orange (Fu et al., 2017); thus these findings provide valuable information for future studies on the role of MTPs in Cu detoxification. Nevertheless, we also found that some *BrrMTP* genes responded to Mg and Na, which have not been used as substrates for MTP transporters in plants either (Kolaj-Robin et al., 2015). Thus, whether our results are related to the special evolution, domestication, or adaptation process of turnips remains unknown. The reasons and mechanisms for these questions need further studies.

Overall, the expression responses to common metal ions of all identified *MTP* family genes in turnip were explored for the first time in the present study. Our results were partly consistent with those of previous studies (Zhang et al., 2013; Fu et al., 2017). Thus, we provided some original information for the subsequent research of several MTP members that have been seldom studied. However, it is incredible to predict the substrate ions for transporters only from the gene expression response, as ion interaction (e.g., antagonism and synergism) or some physiological or metabolic processes affected by certain ion may indirectly cause changes in other ions and the expression dynamic of their corresponding transporters. Thus, concrete gene function studies by modern molecular biology technology are urgently required for each *BrrMTP* gene member.

## Conclusion

We identified 18 candidate *CDF* genes from the turnip genome and presented a comparative genomic analysis of CDFs (or MTPs) in turnip, particularly their phylogenetic relationships, gene structures and chromosome distributions, conserved domains, and motifs. The *CDF* gene family in turnip generated expansions in *MTP1*, *MTP7*, *MTP8*, *MTP10*, and *MTP11* in contrast to those of *A. thaliana*, although *MTP3* might be lost during the evolutionary process. Based on the constructed phylogenetic tree, we divided the BrrMTPs into seven groups (groups 1, 5, 6, 7, 8, 9, and 12), which formed three major clusters (Zn-CDFs, Fe/Zn-CDFs, and Mn-CDFs). The BrrMTP proteins showed similar structural characteristics within groups but significant differences among different groups. Afterward, we performed an expression analysis on all *BrrMTP* members under eight metal treatments (Mg, Zn, Cu, Mn, Fe, Co, Na, and Cd) to investigate the potential roles of BrrMTPs in turnip. The results showed that the expression levels of all *BrrMTP* genes can be induced by at least one metal ion, indicating that these genes may be related to the tolerance or and transport of corresponding metal ions. We found first-hand that two metal ions, namely, Mg and Na, significantly induce the expression levels of several *BrrMTP* genes. According to the different roles of these metal ions for plants, we hypothesized that the *BrrMTP* family genes are possibly involved in heavy metal accumulation and tolerance to salt stress apart from their role in the maintenance of mineral nutrient homeostasis in turnip. However, these conclusions need further verification by concrete gene function analysis.

## Author Contributions

YpY and YhY conceived and designed the experiments. XL, YW, and WH analyzed the data. YW, XL, and BL performed the experiments. XL wrote the manuscript.

## Conflict of Interest Statement

The authors declare that the research was conducted in the absence of any commercial or financial relationships that could be construed as a potential conflict of interest.

## Footnotes

1 https://www.ncbi.nlm.nih.gov/

2 http://www.bioinformatics.nl/brassica/index.html?data=bras_tp%2Fdata&loc=A01%3A11420701..17130662&tracks=DNA&highlight=

3 http://www.ebi.ac.uk/InterProScan/

4 http://gsds.cbi.pku.edu.cn/

5 https://github.com/CJ-Chen/TBtools

6 http://pfam.xfam.org/search#tabview=tab1

7 http://meme-suite.org/tools/meme

8 http://www.cbs.dtu.dk/services/TMHMM/

9 http://web.expasy.org/protparam/

10 https://www.ncbi.nlm.nih.gov/tools/primer-blast/

## Abbreviations

CDF: cation diffusion facilitator
GRAVY: grand average of hydropathicity
His: histidine
MTP: metal tolerance protein
MW: molecular weight
pI: isoelectric point.
