# Supplementary material for: Genome-Wide Identification and Expression Analysis of the Cation Diffusion Facilitator Gene Family in Turnip Under Diverse Metal Ion Stresses
Source: Front Genet. 2018 Apr 4;9:103. doi: 10.3389/fgene.2018.00103 (PMC5893799; doi:10.3389/fgene.2018.00103)
Supplement: Supplementary file 1 [file Table_1.DOCX]

Supplementary Material

Genome-Wide Identification and Expression Analysis of the Cation Diffusion Facilitator Gene Family in Turnip under Diverse Metal Ion Stresses

Xiong Li, Yuansheng Wu, Boqun Li, Wenqi He, Yonghong Yang*, Yongping Yang*

*** Correspondences:** Y. P. Yang: [yangyp@mail.kb.ac.cn](mailto:yangyp@mail.kb.ac.cn); Y. H. Yang: [yyh831994@163.com](mailto:yyh831994@163.com)

# Supplementary Figures and Tables

## Supplementary Figures


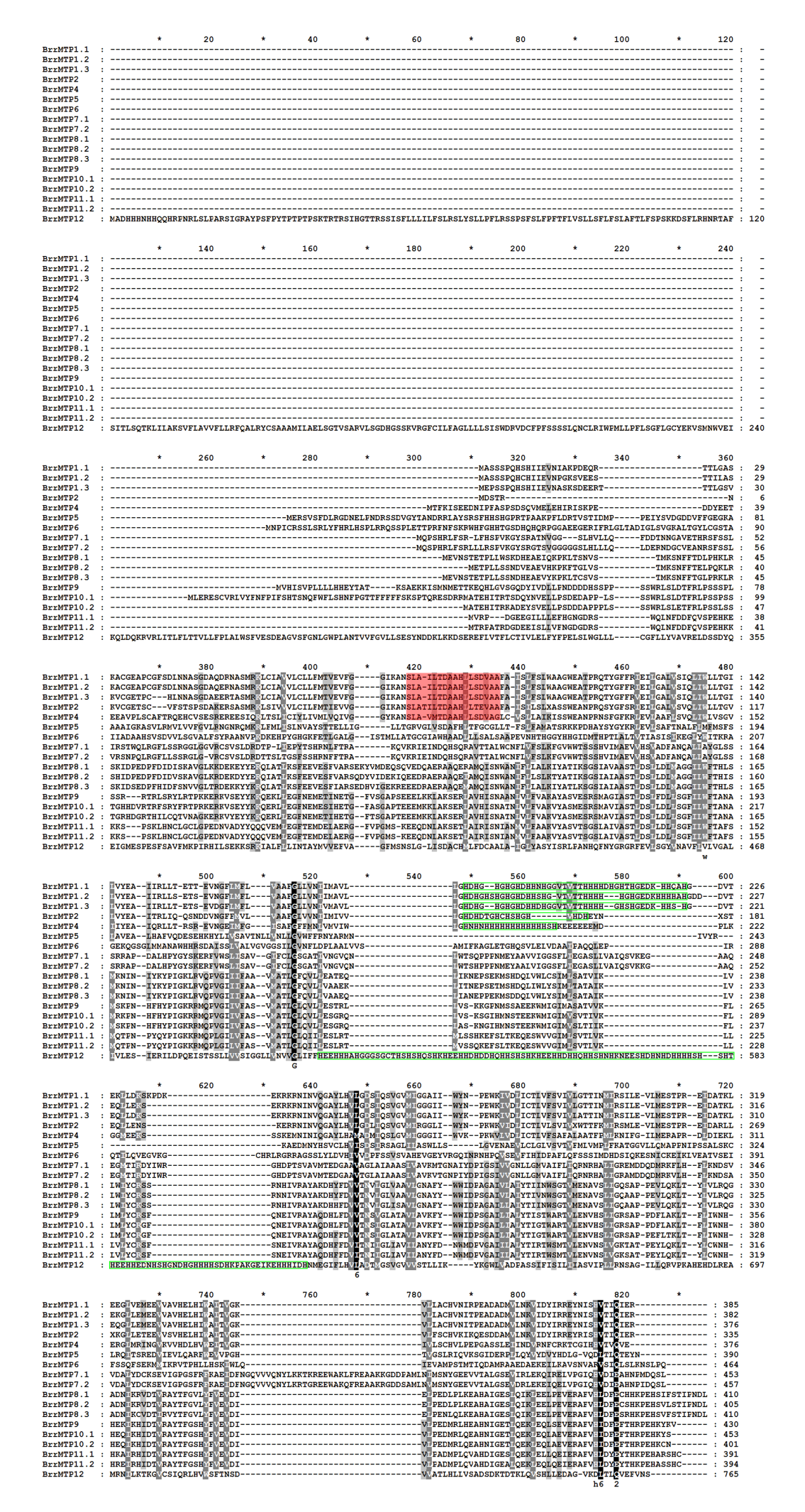


**Supplementary Figure 1** Multiple sequence alignments of the 18 BrrMTP proteins by ClustalW. The black and grey background indicates conserved amino acid residues at different degrees among different sequences. The red region indicates the CDF signature sequence, and the green boxes represent the histidine rich regions.


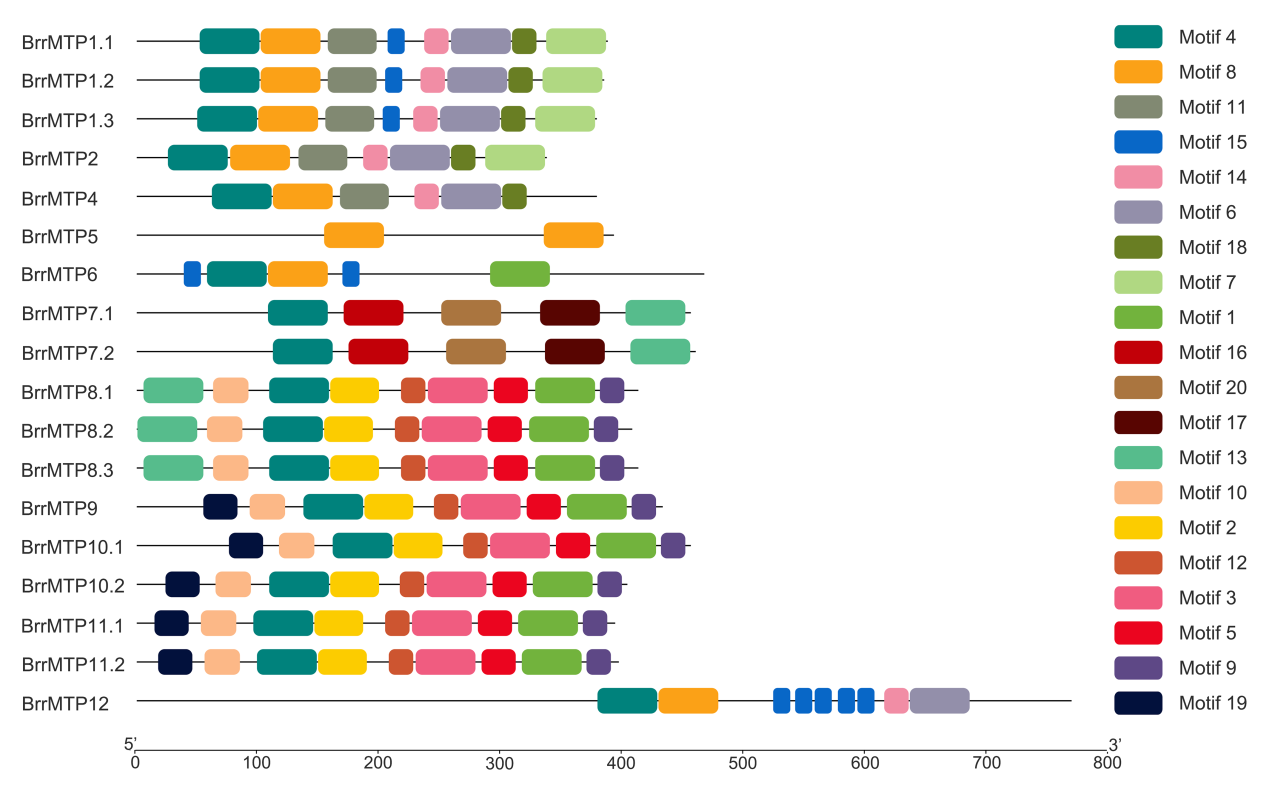


**Supplementary Figure 2** MEME analysis of motif compositions of BrrMTPs. A total of 20 conserved motifs designated as motif 1 to motif 20 were identified within the proteins.

## Supplementary Tables

# Supplementary Table 1 Primer pairs of different *BrrMTP* genes used for qRT-PCR analysis.

| Gene name | Forward primer (from 5’ to 3’) | Reverse primer (from 5’ to 3’) |
| --- | --- | --- |
| BrrMTP1.1 | TAATGGCTGTTCTGCTCGGG | CTTGGTGGTGCTTGTCCTCT |
| BrrMTP1.2 | TCATCGAGGTCAATCCAGGC | AGCTGCGTCGGTGAGTATAG |
| BrrMTP1.3 | TCAAGCCCCCAACATAGTCA | ATAGCACCACAGCGATGCAG |
| BrrMTP2 | TCTCCAAGCGATGCAAAGGA | GCCTTAATGCCACCGACAAC |
| BrrMTP4 | AAGACAATATCCCCTTCGCCTC | GCAAGACAGAGGAACGGCT |
| BrrMTP5 | CTCTGTCGACGGGGATGATG | AGTACGCCACGTTAAGCGAA |
| BrrMTP6 | GGACTGGAAACGGGACATCA | TCCTTCCCCTTAGCCGATGA |
| BrrMTP7.1 | CATCGCATCGCCTCTTCTCT | CAGCTCCGTTGTTTGTGTCG |
| BrrMTP7.2 | GTGAAGGGTTACAGCAGGGG | TAGCTTCCACACACCCATCG |
| BrrMTP8.1 | CGTGTTGATACCGTTCGTGC | TGTTCCGGTTTGTGATGGCA |
| BrrMTP8.2 | ACCGAAGTTTACGGGTTTGGT | GGCCTACAGCTTTGGAGACAT |
| BrrMTP8.3 | ACGTCGTCGGTCTTACTAGAG | CCCAGTTGGAGATTTGCATGG |
| BrrMTP9 | GCCTATGCACAAGACCACCT | TGTGTTCGGGACGATGAGTG |
| BrrMTP10.1 | CCAGTTTTGGTTTCTCTCTCACA | CGTAGCCATTCTCCGATCACT |
| BrrMTP10.2 | TCATCATCGTGGCGTCTCAG | ACGTTCCTTTCCTGCATTGAC |
| BrrMTP11.1 | TCTATGCCTCCGTCACAAGC | TGTTTGCATAGAGAAGGCGGT |
| BrrMTP11.2 | CGCTGGGAATCCTGGTCTTT | AGAGCATGATCCCAACCACC |
| BrrMTP12 | TGGGGAGTGTTGGAGTTGTG | GCAACGGAATGACTGAAGCG |
